# Ventilator touchscreen as source of ESBL-producing *Klebsiella pneumoniae* outbreak

**DOI:** 10.1186/1753-6561-5-S6-O78

**Published:** 2011-06-29

**Authors:** A Narciso, L Gonçalves, A Costa, A Godinho, F Fernandes, A Duarte

**Affiliations:** 1 iMed.UL, Faculdade de Farmàcia, Portugal; 2 SAMS Hospital, Lisboa, Portugal

## Introduction / objectives

*Klebsiella pneumoniae* strains producing extended-spectrum β-lactamases(ESBLs) are a major problem in many different hospitals worldwide,causing outbreaks.

## Methods

An outbreak of ESBL-producing *K. pneumoniae* occurred from 17 August to 28 December 2009 in a acute care hospital, Lisboa. Four patients in intensive care unit were infected or colonized and strains were isolated predominantly from blood and catheter. All infections or colonization were nosocomially acquired, with the patients having been hospitalized from 11 to100 days prior to isolation of the organism. A descriptive and prospective surveillance was performed to control the outbreak and environmental investigations were carried out to identify the source, mainly on equipment for monitorization and medical support.

## Results

Eighteen *K. pneumoniae* strains were identified from patients: seven and nine strains from clinical and screening specimens respectively; and two strains from ventilator touchscreen and suction device manometer. M13 fingerprint analysis revealed closely related strains confirmed by MLST (ST15) performed in selected strains. All of the *K. pneumoniae* isolates had the same pattern of multiresistance.Molecular experiments revealed that CTX-M-15and SHV-28 were the prevalentESBLs. The outbreak was controlled and eliminated by a combination of intensive infection control measures and rigorous local surveillance. These safeguards remain in place and no outbreaks were detected since January 2010.

## Conclusion

To our knowledge, this is the first reported hospital outbreak that provides evidence that the ventilator touchscreen can be a transmission vector for ESBL *K. pneumoniae* isolates.

## Disclosure of interest

None declared.

